# THE LFDSS DETECTS HIGH-RISK FINANCIAL DECISION-MAKING IN COGNITIVELY VULNERABLE OLDER ADULTS

**DOI:** 10.1093/geroni/igz038.457

**Published:** 2019-11-08

**Authors:** Rebecca J Campbell, Peter Lichtenberg

**Affiliations:** Wayne State University, Detroit, Michigan, United States

## Abstract

The Lichtenberg Financial Decision-Making Screening Scale (LFDSS) is a brief screening tool that quantifies an individual’s informed decision making and risk for exploitation in a real-world financial transaction. Previous literature found that the ideal cut-off score for those at higher risk of financial exploitation was five and above. The purpose of the present study was to examine the utility of the LFDSS as a screening tool for financial decision-making ability in a high-risk sample of people with cognitive impairment. The sample was obtained from the Michigan Alzheimer’s Disease Center and was comprised of both cognitively healthy individuals (n=73) and those with cognitive impairment (n=45). All participants completed the LFDSS as part of a larger test battery. A Pearson chi-square analysis was used to examine group differences in financial decision-making risk between cognitively healthy individuals and those with cognitive impairment. A Pearson chi-square analysis found that those who had cognitive impairment were significantly more likely to score above the cut-off for high-risk financial decision-making compared to those who were cognitively healthy (χ2(1)=4.61, p=.032). The base rate of high-risk financial decision-making was 3.2x greater for those with cognitive impairment compared (17.7%) to those who were cognitively healthy (5.5%). These results demonstrate the utility of the LFDSS to detect high-risk decision-making in individuals with cognitive impairment. This tool serves a need in many professional settings (e.g. doctor’s offices and Adult Protective Services) for a brief, standardized assessment measure of financial decision-making and exploitation risk for a real-world, significant financial transaction.

